# Antibody-drug conjugates: the clinical development in gastric cancer

**DOI:** 10.3389/fonc.2023.1211947

**Published:** 2023-05-25

**Authors:** Yingze Zhu, Miao Zhou, Wenyue Kong, Congling Li

**Affiliations:** ^1^ School of Clinical Medicine, Affiliated Hospital, North China University of Science and Technology, Tangshan, China; ^2^ Tangshan Central Hospital, Tangshan, China

**Keywords:** antibody-drug conjugates, gastric cancer, mechanism, therapy, trial

## Abstract

Gastric cancer (GC) is a prevalent malignant tumor of the digestive system worldwide, ranking among the top five in terms of incidence and mortality. However, the clinical efficacy of conventional treatments for gastric cancer remains limited, with a median overall survival of approximately eight months for advanced cases. In recent years, researchers have increasingly focused on antibody-drug conjugates (ADCs) as a promising approach. ADCs are potent chemical drugs that selectively target cancer cells by binding to specific cell surface receptors with antibodies. Notably, ADCs have demonstrated promising results in clinical studies and have made significant strides in the treatment of gastric cancer. Currently, several ADCs are under investigation in clinical trials for gastric cancer patients, targeting various receptors such as EGFR, HER-2, HER-3, CLDN18.2, Mucin 1, among others. This review offers a comprehensive exploration of ADC drug characteristics and provides an overview of the research progress in ADC-based therapies for gastric cancer.

## 1 Introduction

Gastric cancer (GC) is ranked among the top five malignancies worldwide in terms of incidence and mortality, and it is one of the third most common malignancies in China (1). Patients diagnosed with advanced gastric cancer face a grim prognosis, with a median survival of approximately eight months (2). Currently, GC encompasses two histopathological subtypes, namely intestinal and diffuse types, as classified by the Lauren typing system. The intestinal type of GC is primarily linked to H. pylori infection-induced intestinal metaplasia and exhibits a tubular or glandular structure. On the other hand, the diffuse type of GC typically presents with poorly differentiated tumor cells lacking mucosity, infiltrating the gastric tissue as individual cells or small subpopulations (3). Although these histopathological differences have implications for GC prognosis, they do not form the basis for determining treatment options for the disease.

A combination of treatment modalities, including radiotherapy, chemotherapy and immune checkpoint inhibitors, represents the standard approach for various stages of gastric cancer. Unfortunately, the majority of GC patients are diagnosed at an advanced stage (4). Nevertheless, a considerable proportion of GC patients do not receive second-line treatment due to increased adverse effects, inferior treatment outcomes, and reduced tolerability. Traditional chemotherapy regimens are effective in killing tumor cells but often inflict substantial harm on normal cells. Consequently, minimizing off-target toxicity and collateral damage while achieving notable chemotherapeutic efficacy has emerged as a significant hurdle in the management of gastric cancer.

In the early 20th century, the German immunologist Paul Ehrlich proposed the “golden bullet” theory, which suggested the potential of monoclonal antibodies to specifically target and eliminate cancer cells by binding to antigens. The development of antibody-drug conjugates (ADCs) gained momentum in the 1990s, thanks to advancements in chemical linkage technologies and the production of humanized monoclonal antibodies, ultimately leading to their approval for clinical trials (5). In 2000, the first ADC drug, Mylotarg, consisting of a novel anti-CD33 monoclonal antibody combined with calicheamicin, received FDA approval for the treatment of acute myeloid leukaemia (6). However, due to severe off-target effects, the product was later withdrawn from the market in 2010. Recent years have witnessed significant progress in pharmacokinetics and payload potency, enabling ADCs to demonstrate high effectiveness against proliferating tumor cell lines (7). In 2013, the FDA approved KADCYLA as the first ADC drug for the treatment of solid tumors. To date, numerous ADC products have received approval from both the FDA and China’s National Medical Products Administration (NMPA) (8). Distinguished from conventional therapies, ADCs employ linker coupling to combine monoclonal antibodies with cytotoxic payloads (9). This targeted approach reduces damage to normal cells and minimizes systemic toxicity. Looking ahead, ADCs hold great promise as potential new treatment options for cancer patients, including those with gastric cancer.

ADCs, which involve the conjugation of monoclonal antibodies with cytotoxic drugs, represent a novel and promising class of biopharmaceutical compounds in the filed of oncology. Currently, there are 11 FDA-approved ADC drugs and 79 Phase I studies underway, demonstrating their increasing significance. In addition to their proven efficacy and drug toxicity profiles, ADCs exhibit remarkable versatility across various tumor types. Notably, encouraging outcomes have been observed with ADCs such as trastuzumab lutixan (T-DXd) and trastuzumab entansine (T-DM1) in HER2+ breast cancer patients (10). It is worth mentioning that gastric cancer exhibits a higher prevalence of heterogeneous HER2 expression compared to breast cancer (11, 12). This review aims to present the most recent advancements in ADC-related drugs and clinical data specific to gastric cancer, address the current challenges and unresolved issues, and provide valuable insights and future perspectives in this filed.

## 2 Structure and mechanism of ADCs

ADCs consist of three fundamental components, and a thorough comprehension of the drug’s mechanism of action serves as the foundation for their rational design.

### 2.1 Components

**Figure 1:**
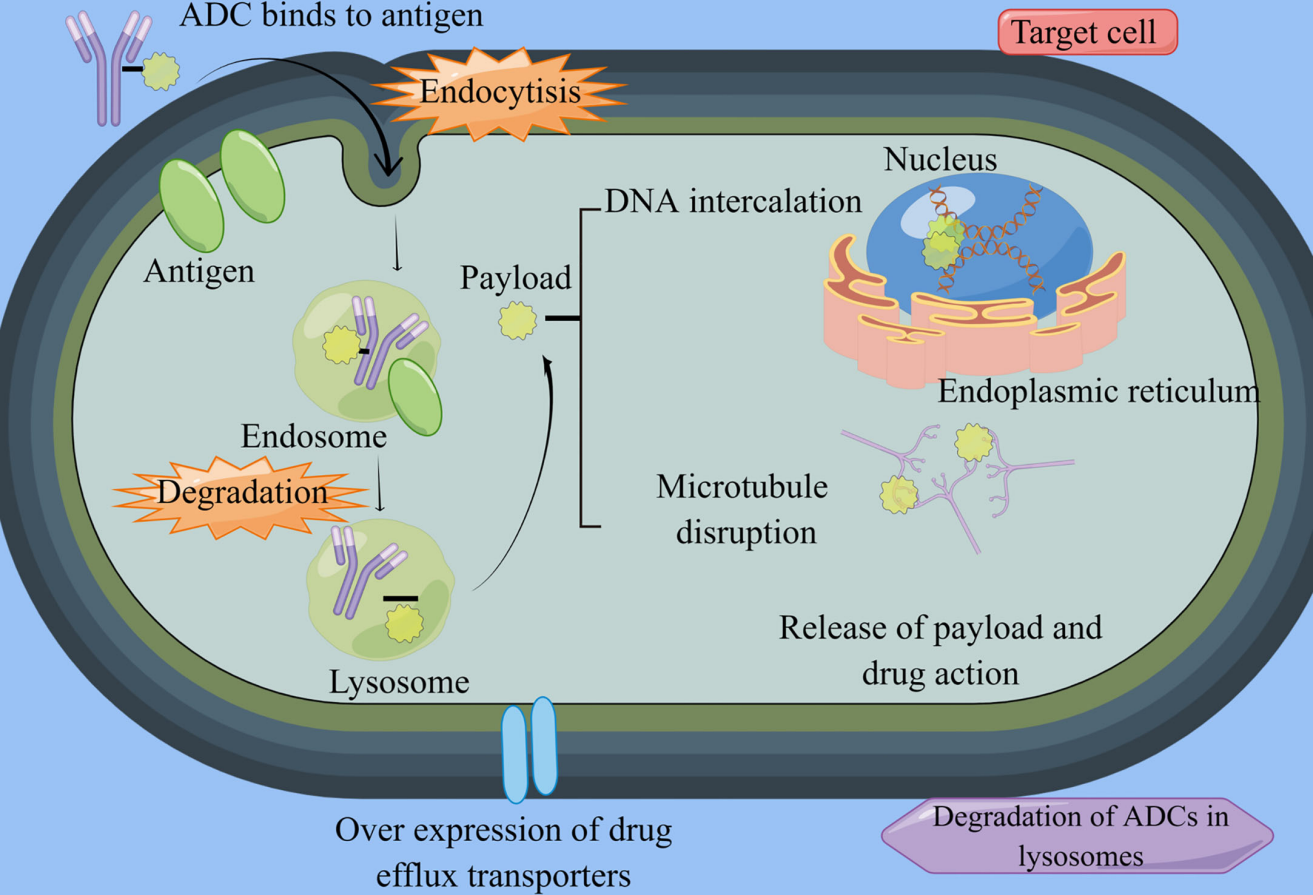
ADCs structure and mechanism. By Figdraw (www.figdraw.com) ADC, antibody-drug conjugate. ADCs consist of antibodies, linkers, and potent cytotoxic agents. Once they enter the bloodstream, these drugs bind specifically to their target cells and undergo endocytosis. Within the lysosomes, the linker is cleaved by cellular proteases, leading to the release of the cytotoxic drug. This drug then acts by disrupting the DNA and microtubules of cancer cells, ultimately inducing apoptosis in the targeted cancer cells.

ADCs represent an innovative class of targeted biotherapeutics that combine monoclonal antibodies with cytotoxic drugs. They primarily consist of antibodies directed against tumor-associated antigens, linkers, and cytotoxic payloads (13) (**Figure 1**). The antibody component exerts its anti-tumor effects by specifically recognizing the antigen on the target cells, facilitating the formation of an antigen-antibody conjugate (14). Following conjugate formation, the cytotoxic payload is rapidly internalized, leading to the release of cytotoxic drugs. The linker component plays a crucial role in connecting and delivering the payload, ensuring a strong attachment of the toxic structure to the antibody, thus minimizing off-target toxicity (15, 16).

Among the aforementioned components, the payload is the most crucial element of ADCs. Studies have indicated that each antibody typically carries an average of 2-4 payload molecules, which is known as the drug-to-antibody ratio (DAR). In general, ADCs with higher DAR values are metabolized more rapidly, a characteristic that largely influences the drug’s therapeutic efficacy. Additionally, the DAR value, PK, and stability are instrumental in determining the size of the conjugation site and the coupling location. Currently, the most extensively studied payloads are DNA damaging agents, DNA transcription inhibitors, and microtubulin inhibitors. Yaghoubi et al. (17) showed that DNA damaging drugs primarily impede tumor cell proliferation through alkylation induced within the DNA double helix groove, nucleic acid strand breakage, or cross-linking reactions. DNA damaging agents include inhibitors such as Amatoxin and Quinolinealkaloid (SN-38). The latter category includes metanephrines and auristatin. Furthermore, RNA splicing inhibitors and RNA polymerase inhibitors are currently under rigorous investigation, with the anticipation of future related drugs for oncology patients, including those with GC.

### 2.2 Si-RNA

In recent years, antibody-siRNA coupled drugs (ARCs) have gained significant attention in scientific research. The previously mentioned ADCs have provided benefits to numerous GC patients by leveraging the target specificity and chemotherapeutic properties of antibodies. However, therapeutic efficacy of ADCs is often constrained by their relatively low drug delivery capacity at safe dosage levels, which restricts the amount of chemotherapeutic agent that reaches the intended target area. In contrast, ARCs serve as promising carriers for siRNA drug delivery, enabling precise and effective targeting of cancer cells (18).

The primary mechanism of action of anti-siRNA drugs lies in their ability to inhibit the expression of target genes through RNA interference (RNAi). Ribonucleic acid endonucleases can produce siRNAs, each containing approximately 22 bases, by cleaving short hairpin RNA (shRNA) and longer double-stranded RNA (dsRNA) (19). The stability and efficacy of siRNA molecules can be enhanced through chemical modifications to the phosphoglycan backbone and its constituent pyrimidine and purine bases. For example, replacing the unstable phosphodiesterase backbone with a phosphorothioate backbone improves albumin binding by enhancing the hydrophobicity of the molecule. This alteration effectively reduces the drug’s degradation rate *in vivo*, prolongs the circulation time, and enhances the pharmacokinetics (20). In recent years, the FDA has approved several siRNA drugs, including Givosiran, Patisiran, Inclisiran, and Lumasiran (21, 22). Notably, Givosiran has significant implications in liver-related conditions. The drug achieves good therapeutic effects through a GalNAc-directed siRNA delivery system (23). Despite the promise of ARC drugs, they encounter a series of challenges, such as the difficulty of cellular uptake due to the inherent negative charge of siRNA, the endocytosis of the target antigen, and the lack of quantitative methods to study siRNA endosomal escape. The development and application of many ARC drugs are currently in their preliminary stage. Further in-depth research is needed to pave the way for a new chapter of ARC therapies.

### 2.3 Mechanism of drug resistance

While ADCs are currently offering significant benefits to gastric cancer patients, issues related to resistance mechanisms for each component are surfacing. These currently include downregulation of cell surface antigens like HER2, drug efflux proteins, impaired endocytosis, defects in internalization pathways such as AKT signalling, overexpression of transporters such as V-ATP binding cassettes, and lysosomal degradation (24–26).

ADCs exhibit a unique resistance mechanism, specifically involving defective lysosomal degradation or an internalization pathway. The degradation of ADCs within lysosomes depends on environmental conditions and enzymatic activity. In T-DM1-resistant gastric cancer cells, researchers have observed lysosomal alkalinization or reduced lysosomal protein hydrolase activity (27). Wang et al. (28) showed that the internalization and externalization kinetics of T-DM1 in gastric cancer cells were similar between parental and resistant cells. However, in N87 gastric cancer cells exhibiting drug resistantance, aberrant V-ATPase was found to impact T-DM1 proteolysis, leading to significant changes in drug resistance. Furthermore, Sung et al. (29) demonstrated in a HER2+ cancer model that when N87-TM cells were implanted into athymic mice, they could modulate internalization and transport pathways through the mediation of related factors. This mechanism is fundamentally associated with T-DM1 resistance. The researchers developed a T-DM1-resistant cell model using a circulatory route of administration and analyzed differences in membrane fractions through proteomic techniques. Overexpression of transcript release factor, caveolin-1, and polymerase I affected caveolin-mediated endocytosis in this cell model. Unfortunately, the knockdown of caveolin-1 did not appear to significantly influence the restoration of T-DM1 sensitivity.

At present, the investigation into the resistance mechanism of ADCs in gastric cancer has been limited to pre-clinical models, and its broader application in future clinical treatments remains to be seen.

## 3 Targets and antibody-drug conjugates

**Figure 2:**
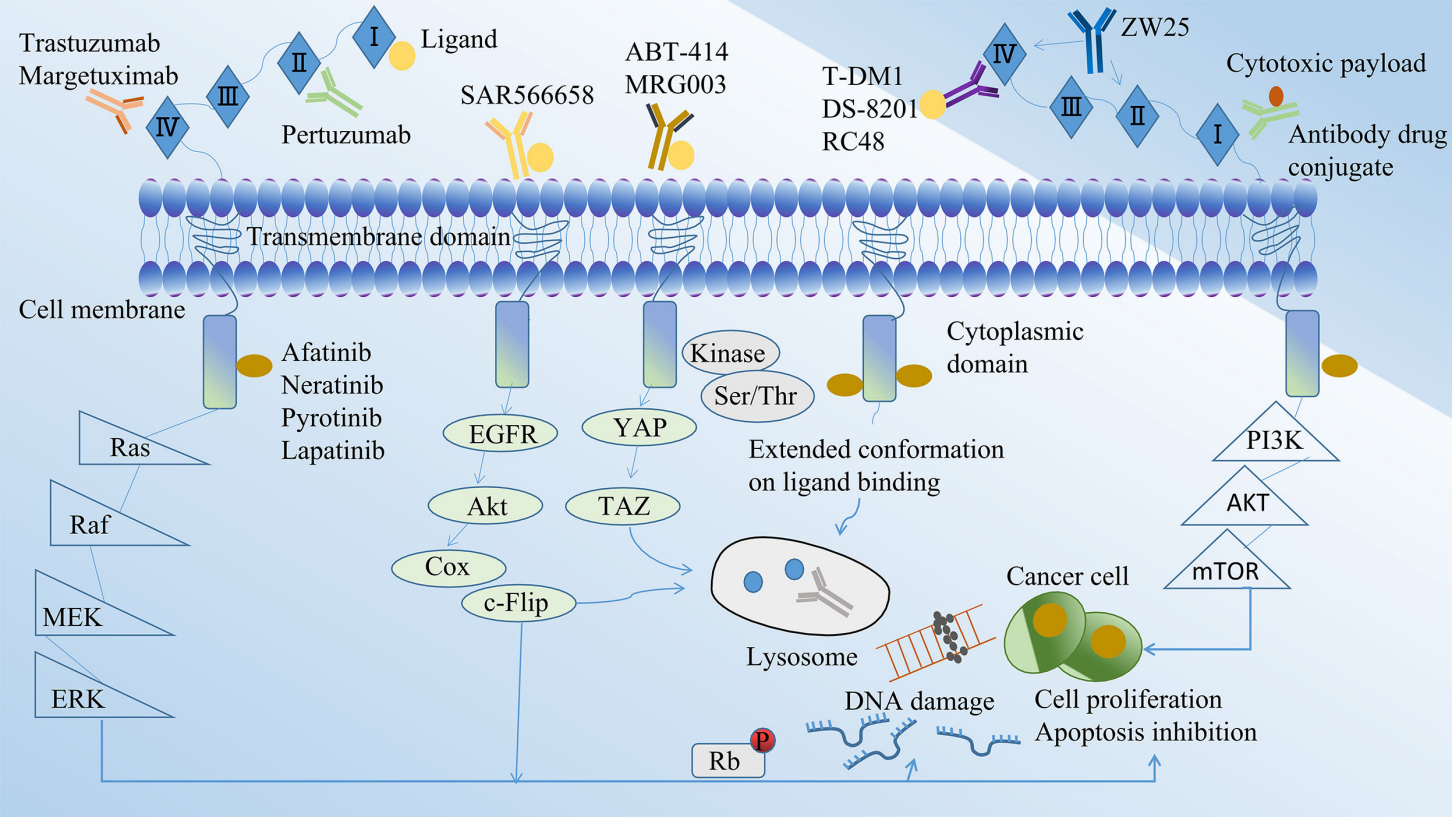
Mechanisms for the study of ADCs in gastric cancer.

**Figure 3:**
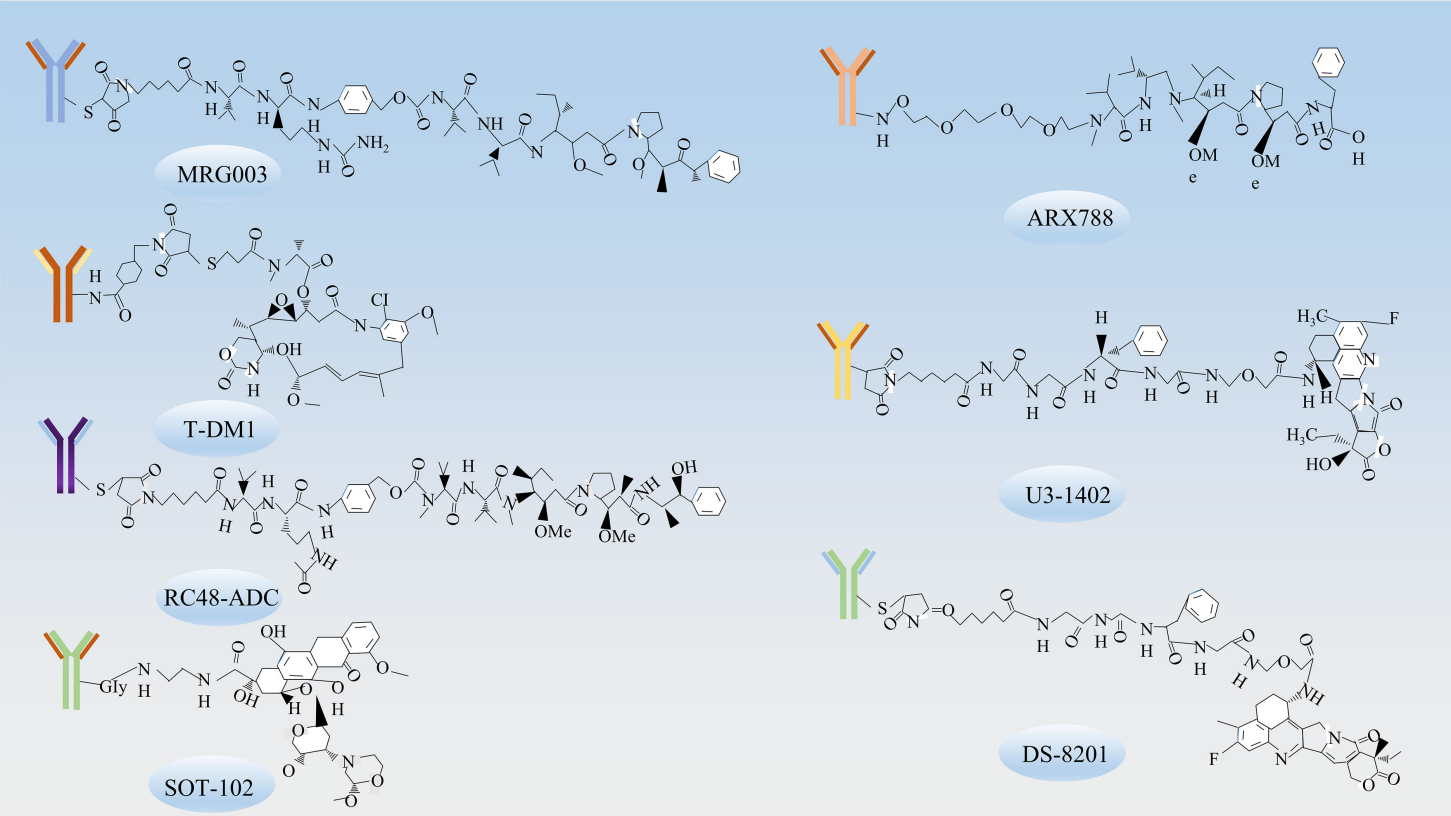
The chemical structures of ADCs. The chemical structures of ADCs targeting various receptors include MRG003, which targets EGFR; T-DM1, ARX788, DS-8201, RC48-ADC, which target HER-2; U3-1402, which target HER3; and SOT-102, which targets CLDN18.2.

Research on ADCs in gastric cancer treatment is burgeoning as therapeutic strategies continue to advance (**Table 1**). In recent years, therapeutics targeting molecules such as EGFR, HER-2, HER3, CLDN18.2, Mucin 1 have shown a degree of efficacy in managing progressive gastric cancer (**Figure 2**) (**Table 2**). The chemical structures of these drugs can be viewed in **Figure 3**.

**Table 1:** ADCs involving in clinical trials on cancers treatment.

Trial	Phase	Patients (n)	Drugs	ORR,% (95% CI)	mPFS, months (95% CI)	mOS, months (95% CI)	Ref.
KSCC/HGCSG/CCOG/CCOG/PerSeUS 1501B	II	42	T-Mab	82.1 (95% CI 70.0 -90.0)	7.0 (95% CI 5.5-14.1)	27.6 (95% CI 15.6-NR)	(30)
GATSBY	II/III	415	T-DM1	20.6 (95%CI 15.3-25.8)	2.7 (95%CI 1.61-2.79)	7.9 (95%CI 6.7-7.9)	(31)
			PTX	19.6 (95%CI 13.7-25.5)	2.9 (95%CI 2.76-4.01)	8.6 (95% CI 7.1-11.2)	
NCT02564900	I	54	T-DXd	37 (95%CI 24.3-51.3)	11.1 (95% CI 7.6-NR)	29.4 (95% CI 12.9-29.4)	(32)
DESTINY-Gastric01	I	187	DS-8201	51.3 (95% CI 41.9-60.5)	5.6 (95% CI 4.3-6.9)	12.5 (95% CI 10.3-15.2)	(33)
			Chemotherapy	14.3 (95% CI6.4-26.2)	3.5 (95% CI 2.0-4.3)	8.4 (95% CI 6.4-10.4)	
DESTINY-Gastric02	II	79	DS-8201	38 (95% CI 27.3-49.6)	5.6 (95% CI4.2-8.3)	12.1 (95% CI 9.4-15.4)	(34)
NCT02881190	I	57	RC48	23.6 (95% CI 18.3-28.9)	–	12.6 (95% CI 9.8-15.6)	(35)
RC48-C008	II	125	RC48-ADC	18.1 (95%CI 11.8-25.9)	3.8 (95% CI 2.7-4.0)	7.6 (95% CI 6.6-9.2)	(36)
CTR20190639	I	30	ARX788	37.9 (95% CI 20.7-57.7)	10.7 (95% CI: 4.8-NR)	4.1 (95% CI 1.4-6.4)	(37)
NCT04868344	I	39	MRG003	75 (95%CI 50.9-91.3)	2.8 (95%CI 1.2-4.1)	11.8 (95%CI 3.4-11.8)	(38)

**Table 2:** Drugs targeting different targets.

ADC	mAb	Payload	Linker	DAR	Approved	Company	Year
Trastuzumab Emtansine (T-DM1)	Trastuzumab	DM1	Non-cleavable SMCC linker	3.5	HER-2	Genentech	2013
Trastuzumab Deruxtecan (DS-8201a)	Trastuzumab	Dxd	Cleavable GGFG linker	7-8	HER-2	Daiichi Sankyo	2021
Disitamab Vedotin (RC48)	Aidixi	MMAE	Cleavable vc-PABC linker	4	HER-2	RemeGen	2021
Trastuzumab Duocarmazine (SYD985)	Trastuzumab	Seco-DUBA	Cleavable vc linker	2.7	HER-2	Byondis	2017
ARX-788	Anti-HER2 mAb (ARX269)	MMAF	Non-cleavable linker conjugated to pAcF	1.9	HER-2	Ambrx	2021
A166	Anti-HER2 mAb	Duostatin-5	Cleavable vc linker	N/A	HER-2	Klus	2021
MRG002	Anti-HER2 mAb	MMAE	Cleavable vc linker	3.8	HER-2	Miracogen	2020
ALT-P7	Trastuzumab biobetter (HM2)	MMAE	Cleavable cysteine-containing peptide linker	2	HER-2	Alteogen	2020
GQ1001	Trastuzumab	DM1	N/A	N/A	HER-2	GeneQuantum	2022
SBT6050	Anti-HER2 mAb	Toll-like receptor 8 agonist	N/A	N/A	HER-2	Silverback	2020
PF-06804103	Trastuzumab-derived Ab	Aur-0101	Valine-citrulline linker	4	HER-2	Pfizer	2020
Gemtuzumab Ozogamicin (MYLOTARG)	Gemtuzumab	Ozogamicin/Calicheamicin	Acid cleavable linker	2-3	CD33	Pfizer	2017
Brentuximab Vedotin (ADCETRIS)	Brentuximab	MMAE	Enzyme Cleavable linker	4	D30	Seagen	2011
Inotuzumab Ozogamicin (BESPONSA)	Inotuzumab	Ozogamicin/ Calicheam-icin	Acid cleavable linker	6	CD22	Pfizer	2017
Polatuzumab Vedotin (POLIVY)	Polatuzumab	MMAE	Enzyme Cleavable linker	3.5	CD79 b	Roche	2019
Enfortumab Vedotin (ADCEV)	Enfortumab	MMAE	Cleavable linker	3.8	Nectin4	Seattle Genetics	2019
ABT-414	Anti-EGFR mAb	MMAF	Non-cleavable mc linker	4	EGFR	AbbVie	2020
MRG003	Anti-EGFR mAb	MMAE	Non-cleavable mc linker	N/A	EGFR	Miracogen	2020
M1231	Anti-EGFR mAb	Hemiasterlin	N/A	N/A	EGFR	Sutro EMD Serono	2021
Sacituzumab Govitecan (TRODELVY)	Sacituzumab	Govitecan	Cleavable linker	7.6	Trop2	Immunomedics	2020
Sacituzumab Govitecan (IMMU-132)	Sacituzumab	IMMU-132	N/A	N/A	Trop 2	Immunomedics	2022
SKB246	Pembrolizumab	MMAE	Cleavable linker	7.4	Trop 2	Kelun-Biotech	2022
DS-1062	Datopotamab	DX-8951f	Acid cleavable linker	4	Trop 2	Daiichi Sankyo	2020
SYSA1801	Anti- Claudin 18.2 mAb	MMAE	Cleavable linker	2	Claudin 18.2	CSPC	2021
CMG901	Anti- Claudin 18.2 mAb	MMAE	Cleavable linker	7.4	Claudin 18.2	Keymed	2020
RC118	Anti- Claudin 18.2 mAb	MMAE	Cleavable linker	N/A	Claudin 18.2	Remegen Co.,Ltd	2021
SKB315	Anti- Claudin 18.2 mAb	MMAE	Cleavable linker	N/A	Claudin 18.2	Kelun-Biotech	2021

### 3.1 ADCs targeting EGFR

ADCs targeting EGFR include ABT-414 and MRG003. EGFR, also known as ERBB1, belongs to the ERBB receptor tyrosine kinase family and is a transmembrane protein receptor comprised of an extracellular ligand-binding domain, a transmembrane structural domain, and an intracellular kinase domain.

Depatuxizumab mafodotin (ABT-414) is an ADC that targets EGFR. It consists of the humanized antibody ABT-806, approximately four MMAFs, and a maleimide hexyl group. Notably, ABT-806 exhibits a low binding affinity to EGFR in normal tissues. However, ABT-414 retains the positive functional properties of ABT-806 and shows considerable efficacy in xenograft models (39). Upon specifically recognizing cancer cells with high levels of wild-type EGFR, the drug binds with high affinity to the mutant EGFR VIII, thereby exerting anti-tumor effects by inhibiting the EGFR/ERK pathway. In addition, MRG003, an ADC targeting EGFR, is composed of EGFR-specific IgG1 antibodies, monomethyl orexin E, and linkers. This drug specifically recognizes and binds to EGFR on the surface of tumor cells, subsequently activating the Hippo pathway through tyrosine phosphorylation. Notably, Hpo is a Ser/Thr kinase that binds and phosphorylates the YAP/TAZ regulator (38). Eventually, the protease activity releases the small molecule MMAE, which binds to the intracellular microtubulin and inhibits microtubulin polymerization, disrupting mitosis and various cellular physiological functions, thereby inhibiting tumor cell proliferation and leading to tumor cell death. Currently, Phase II trials such as NCT04838964, NCT04868162, and NCT04868344, focusing on gastrointestinal and head and neck tumors, are ongoing while awaiting the collation of pertinent results. Among the 39 patients enrolled in the NCT04868344 trial, partial remission and disease stabilization rates of 21% and 31%, respectively, were achieved, which attests to the anti-tumor activity of the drug (38).

### 3.2 ADCs targeting HER-2

The HER-2/neu protein plays integral roles in a multitude of cellular functions including, but not limited to, cell proliferation, apoptosis, migration, and angiogenesis (40).

#### 3.2.1 T-DM1

T-DM1 is a compound consisting of trastuzumab, a microtubulin inhibitor called DM1, connected by a non-cleavable, non-reducing thioether linker with a DAR of 3.5 (41). It was developed by Roche’s team (42) and received approval from the FDA for the treatment of HER2-positive advanced breast cancer (43). This approval laid the foundation for further studies of T-DM1 in the context of gastric cancer. T-DM1 operates as a targeted delivery system, transporting DM1, an apoptosis inducer, to HER2-positive tumor cells, thereby functioning as a “preparative missile”. Trastuzumab, one of the components of T-DM1, binds to the extracellular structural domain IV of the HER2 factor, disrupting the HER2/HER3 interaction in cells that overexpress HER2 and affecting the MAPK-RAS/RAF signalling pathway (31). The drug hampers tumor cell growth by upregulating the expression of the cell cycle inhibitor protein p27, inducing cell cycle arrest during the G1 phase in tumor cells. In preclinical trials, T-DM1 exhibited potent anti-GC cell proliferation properties. However, it did not demonstrate a significant survival benefit in subsequent clinical trials (44). The GATSBY trial, a randomised, open, multicentre phase II/III clinical study, evaluated the effectiveness and tolerability of T-DM1 in HER2-positive gastric cancer patients following treatment progression. The median overall survival was 7.9 months (95% CI 6.7-9.5) for the TDM-1 group and 8.6 months (95% CI 0.87-1.51) for paclitaxel treatment group, once again indicating that T-DM1 did not provide a survival advantage (31). The aforementioned clinical trial’s outcomes suggest that gastric cancer is a highly heterogeneous tumor. As a result, large clinical trials are needed to thoroughly investigate the potential of T-DM1 as a viable treatment option for advanced gastric cancer.

#### 3.2.2 DS-8201

DS-8201, an ADC, is composed of trastuzumab, a cleavable peptide linker, and a topoisomerase I inhibitor (DXd) (45).Unlike T-DM1, which has a drug-to-antibody ratio (DAR) of 3.5, DS-8201 boasts a DAR of 8, thereby enhancing its potency even against tumor cells exhibiting low HER2 expression. The elevated DAR of DS-8201 can be attributed to its unique linker, a tetrapeptide sequence (glycyn-phenylalanyn-glycyn, or GGFG), which is selectively cleaved by lysosomal enzymes in tumor cells. This specific cleavage ensures the efficient release of the drug into tumor cells without affecting the surrounding circulation (41). In addition, this ADC drug retains the activity of trastuzumab, inhibiting the PI3K/AKT signaling pathway and HER2 shedding, while triggering antibody-dependent cellular cytotoxicity (ADCC) in combination with the Fcγ receptor (46).

The DESTINY-Gastric01 trial showed a significant increase in ORR (objective response rate) and mOS (median overall survival) in patients treated with DS-8201 compared to conventional chemotherapy, thereby highlighting the survival benefits conferred by ADCs. The findings were further corroborated by the subsequent DESTINY-Gastric02 trial, demonstrating their broad applicability (34, 47). Following these breakthrough findings, a phase I clinical trial conducted by Shitara et al. (10) demonstrated a tolerable safety profile and preliminary anti-tumor activity with T-DXd in patients with HER2-positive progressive gastric cancer. A subsequent phase I trial showed an extended overall survival in the T-DXd group compared to those receiving standard chemotherapy regimens (33). Currently, the FDA has approved DS-8201 for the treatment of patients with unresectable or metastatic HER2-positive gastric cancer. The ongoing DESTINY-Gastric06 trial aims to confirm the survival benefit of DS-8201 in the Chinese patient population. The current standard second-line treatment for patients with advanced gastric cancer is a combination of ramucirumab and paclitaxel. In pursuit of an improved treatment alternative, the DESTINY-Gastric04 trial is being concurrently conducted. Surprisingly, Ogitani et al. (48) found anti-tumor activity at low levels of HER2 in a PDX model of breast cancer. A gastric cancer dose toxicity study, which included patients with low HER2 expression gastric cancer, showed significant anti-tumor activity in a phase I pilot study, thus suggesting the potential efficacy of this therapy in patients with low HER2 expression gastric cancer (32).

#### 3.2.3 RC48

RC48 is an ADC specifically targeting HER2. It comprises monoclonal antibodies Hertuzumab, MMAE and histone B (cathepsin B, cat B)-sensitive cleavable linker mc-vc. This ADC, developed in China, has exhibited a favourable safety profile along with promising anti-tumor activity in gastric cancer studies (35).

RC48 functions by modulating tumor cell proliferation, differentiation, apoptosis, and migration. It achieves this through the activation of the Ras/MAPK signalling pathway via heterodimer formation and tyrosine kinase autophosphorylation-mediated signal transduction (49). The drug interacts with the HER2 antigen present on the surface of tumor cells, subsequently undergoing endocytosis to enter the lysosomal compartment. The drug’s linker is catalyzed by cat B to break and release MMAE. This cytotoxic agent then translocates from the lysosome to the cytoplasm where it binds to microtubule proteins. This interaction inhibits microtubule dynamics, causing cell cycle arrest and ultimately inducing programmed cell death (50). In a phase I trial conducted by Gong et al. (51), RC48 demonstrated notable efficacy in patients with HER2-positive gastric cancer. Further, a phase II study (RC48-C008) involving 125 patients reported an ORR of 18.1% (95% CI 11.8%-25.9%), a mPFS of 3.8 months (95% CI 2.7-4.0), and a mOS of 7.6 months (95% CI 6.6-9.2) (36). In light of these promising anti-gastric cancer effects, the Chinese Drug Administration officially approved RC48 in 2021 for the treatment of locally advanced or metastatic gastric cancer. This marked RC48 as the only domestically-approved ADC drug for gastric cancer in China. Currently, a Phase III confirmatory study (C007, NCT04714190) is in progress, with the medical and scientific communities eagerly anticipating the final results.

#### 3.2.4 ARX788

ARX788 is composed of a humanized anti-HER2 monoclonal antibody with a targeted modification of non-natural amino acid, coupled with a microtubule protein inhibitor MMAF through a stable non-cleavable linker segment (52).

ARX788 offers the advantage of stable linkage as a next-generation site-specific anti-HER2 ADC. The drug exerts its clinical effects by modulating the Ras/MAPK and PI3K/AKT signaling pathways (52). Phase I clinical studies evaluated the impact of ARX788 in 30 patients with advanced or metastatic gastric cancer. The combination of ARX788 and trastuzumab demonstrated a significant improvement in overall survival, prolonging it by 10.7 months, as well as an increase in median progression-free survival by 4.1 months, compared to patients treated with trastuzumab alone (37). In 2021, ARX788 was granted orphan drug status by the FDA for the treatment of HER2-positive gastric cancer and gastroesophageal junction cancer. The ongoing Phase I dose-escalation trial (NCT03255070) is investigating ARX788 in patients with HER2-positive gastric cancer, with the aim of providing stronger evidence to support its clinical criteria.

#### 3.2.5 Others

In addition to the aforementioned targets, there are several other clinical agents that target HER-2, including XMT-1522, MEDI4276, MRGOO2, and TR1801-ADC.

The effectiveness of XMT-1522 against T-DM1-resistant gastric cancer cell lines and xenograft models was demonstrated in cellular and mouse models (26). In the clinical trial NCT02952729, XMT-1522 exhibited low gastrointestinal toxicity with grade 1 or 2 adverse events (53). MEDI4276, a novel ADC, was evaluated in a phase I dose-escalation study involving HER2-positive patients with advanced gastric cancer. The study’s primary endpoints were safety and tolerability, while secondary endpoints included anti-tumor activity. MEDI4276 was well-tolerated at a dose of 0.3g/kg, and it showed evidence of anti-tumor activity (54). This ADC is anticipated to become a new HER2-targeted drug for gastric cancer. Similarly, MRGOO2 and TR1801-ADC demonstrated significant anti-tumor activity in preclinical studies and are currently either in or prepared for phase I clinical trials (55, 56). Additionally, ongoing trials including NCT03262935, NCT04602117, NCT04235101, and NCT01042379 are investigating SYD985. These trials aim to assess the pathological complete response (PCR) of different biologics in combination with chemotherapy, expanding the chemotherapy combination options for ADCs in the first-line treatment of patients with advanced gastric cancer. A Phase I/II clinical study of A166 (NCT03602079) is currently recruiting patients with gastric cancer, and the trial results have not yet been disclosed.

### 3.3 ADCs targeting HER-3

U3-1402 is an innovative HER3-targeted ADC that was developed in Japan (57). It comprises the patritumab monoclonal antibody, the small molecule cytotoxin DXd, and a tetrapeptide linker (58).

The drug exhibited several advantages, including strong targeting, low toxic side effects, long duration of action, and potent activity. It binds to HER3, which is aberrantly expressed on the surface of tumor cells, forming the HER3-U3-1402 complex. This complex is internalized into the endosome and subsequently engulfed by lysosomes. Within the lysosome, the histone protease cleaves and releases the free topoisomerase I inhibitor DXd, leading to DNA damage and cellular regulation (59). In preclinical trials, it demonstrated significantly superior activity compared to the patritumab monoclonal antibody, showing therapeutic efficacy in both first-generation TKI-resistant cell lines and mouse models (60). Data from the phase I clinical trial (NCT03260491) in patients with advanced NSCLC demonstrated 100% disease control with this drug (61). Two ongoing clinical studies evaluating HER3-positive breast cancer have indicated favorable survival benefits, with ORRs of 33% and 42.9%, respectively (62, 63). In summary, U3-1402 exhibits strong targeting, low toxic side effects, long duration of action and potent activity, forming the basis for clinical trials in gastric cancer. It is anticipated to provide new therapeutic options for EGFR-TKI-resistance patients, addressing EGFR-TKI resistant in gastric cancer.

In addition to U3-1402, several other HER3-targeted ADCs are currently being investigated in clinical trials, namely MCLA-128, MM-121, CDX/KTN3379, and GSK-2849330. The outcomes of these trials are anticipated to provide potential benefits to GC patients in the future.

### 3.4 ADCs targeting Claudin 18.2

Claudin 18.2 belongs to the transmembrane family of epithelial tight junction proteins, which are prominently expressed on the surface of gastric cancer tissue.

The first ADC targeting Claudin 18.2 entered clinical trials in 2000. Claudin 18.2 is utilized in differentiated formats for gastric cancer treatment, including monoclonal antibodies, dual antibodies, ADCs, and CAR-T therapies. SYSA-1801, a fully human monoclonal antibody-MMAE drug conjugate, inhibits microtubulin polymerization, leading to effective mitotic inhibition. It has been approved for Phase I clinical trials in advanced gastric cancer (64). Animal experiments have demonstrated the effective targeting of tumor cells by SYSA-1801 through anti-Claudin 18.2 antibodies, resulting in the elimination of gastric cancer cells. Additionally, CMG-901, a Claudin 18.2-targeted antibody-drug conjugate developed by Connoia, is being used for the treatment of patients with GC who have failed or progressed on standard therapy (65). The drug comprises an antibody specific to Claudin 18.2, MMAE, and a cleavable linker. Claudin 18.2 is exposed by gastric cancer cells due to disruption of tight junctions, and CMG-901 plays a crucial role in tumor eradication. The clinical trial application for a Phase I trial of the Claudin 18.2 ADC in GC and gastroesophageal junction cancer received approval in both China and the USA. By 2022, the Phase I clinical study of CMG901 in eight patients with Claudin 18.2-positive gastric or gastroesophageal junction adenocarcinoma reported an objective remission rate of 75% and a disease control rate of 100%, with the determination of mPFS and mOS pending (66). The recommended dose, safety, and tolerability of the drug were established in the NCT05205850 trial involving patients with locally advanced unresectable or metastatic GC and gastroesophageal junction cancer. Besides the aforementioned ADCs, IMAB362 is also undergoing clinical trials. Studies have indicated the drug’s safety and tolerability in GC patients. PFS and mOS in GC patients were extended by 3.1 months and 4.8 months, respectively. Furthermore, phase II clinical trials have demonstrated improved anti-tumor activity in combination with chemotherapeutic agents or as monotherapy (67).

### 3.5 ADCs targeting Mucin 1

Mucin 1 (MUC1) is a significant member of the Mucin family, characterized by its high glycosylation. It is a type I transmembrane protein with a core protein size of 2250 kDa, and it is prominently expressed in the apical region of epithelial cells in various organs, including the gastrointestinal tract, lung, kidney, uterus, and eye.

MUC1-based ADCs target both MUC1 glycosyl-associated proteins and the MUC1 protein backbone (68). Several ADCs have been developed to specifically target MUC1 for the treatment of gastric cancer, demonstrating significant anti-tumor activity (69). In animal tumor models, the effective dosage of SAR566658 correlates with the expression of CA6, a MUC1-associated sialic acid glycoprotein. SAR566658 comprises the anti-CA6 antibody huDS6 conjugated to DM4, including tumor cell death through CA6 recognition (70). In addition to ADCs targeting MUC1 glycosylation-related proteins, the MUC1 protein backbone itself is an important target. Panchamoorthy et al. (71) developed a 3D1-MMAE ADC based on MUC1-C and validated its ability to kill MUC1-C-positive tumor cells *in vitro*. Wu et al. (72) developed a humanized MUC1 antibody called HzMUC1. This ADC combines HzMUC1 with MMAE and demonstrates the inhibition of trastuzumab-resistant, HER2-positive cancer cell growth by inducing G2/M cell cycle arrest and promoting apoptosis.

## 4 ADCs-IO combination therapy

Immuno-oncology (IO) has been approved for the treatment of various cancers, including gastric cancer, non-small cell lung cancer, lymphoma, Hodgkin’s lymphoma, and kidney cancer. It has emerged as one of the most promising areas of research in cancer therapy.

Researchers have made significant progress in understanding the various mechanisms employed by tumor cells to evade the host’s immune response. Immune checkpoint-mediated pathways involving CTLA-4, PD-1 and PD-L1 have emerged as crucial players in immunosuppression. However, the effectiveness of immune checkpoint inhibitors (ICIs) remains limited, with only approximately 30% of patients exhibiting a favorable immune response rate. Patients who demonstrate a better response to checkpoint blockade therapy typically exhibit higher levels of CD8^+^ T cells within their tumors prior to treatment (73). In contrast, studies have shown that certain chemotherapeutic agents and physical irradiation can induce Immunogenic Cell Death (ICD), leading to T cell recruitment and activation of effector T cells within tumors (74). Preclinical evidence suggests that ADCs have the potential to modulate the tumor microenvironment, potentially enhancing the response to detection of ICIs (75). In mouse models, T cells and macrophages in the tumor microenvironment exhibit overexpression of CTLA4 and PD-1/PD-L1, with synergistic effects observed when combining T-DM1 with ICIs (76). Additionally, the combination of HER2-targeted drugs and PD-1 inhibitors has shown synergistic potency (77). Furthermore, studies have demonstrated that ADC payloads can directly induce dendritic cell activation, maturation, and trigger ICD, thereby augmenting the immune response (57, 78). The monoclonal antibodies present in ADC structures may upregulate PD-1/PD-L1 expression levels, enhance immune cell infiltration, promote antigen presentation by dendritic cells, and ultimately improve the efficacy of PD-1/PD-L1 inhibitors. Currently, Atezolizumab, Nivolumab, and Pembrolizumab are among the most commonly used ICIs in combination with ADCs. However, clinical studies investigating their efficacy in gastric cancer treatment are still at an early stage and require further research in the future.

## 5 Limitations of ADCs

ADCs, similar to other anti-cancer drugs, provide survival benefits to patients. However, they are not without challenges, as they can encounter resistance issues influenced by multiple factors. To enhance drug efficacy, overcome resistance, and optimize their composition, further advancements are necessary.

### 5.1 Antigenic ligand factors

Upon administration, the drug selectively attaches to the tumor cell surface antigen, prompting endocytosis of the ADCs by the cells. In some instances, ADCs can also bind to the Fc receptor within the body. Any disruption to this process can impact the cells’ responsiveness to ADCs, thereby facilitating the emergence of drug resistance.

The presence of neuromodulin, a ligand for ErbB3, leads to heterodimerization of ErbB2, ErbB3, and ErbB4, thereby reducing the cytotoxicity of T-DMI in gastric cancer cell lines (79). Once the target antigen is bound, ADCs-antigen complexes undergo internalization through niche or lattice-protein-mediated cytosolic drinking or endocytosis (29). T-DM1 resistant N87 cells internalize ADCs into intracellular fossa proteins and alter their transport to lysosomes. Sung et al. (80) showed that the co-localization of T-DM1 to intracellular niche protein-positive sites in ErbB2-positive cell lines correlated with a diminished response to T-DM1. Within the cell, degradation of ADCs in the lysosome can be impaired by reduced lysosomal acidification or protein hydrolysis, leading to an inability to cleave the cytotoxic payload of ADCs. T-DM1-resistant cell lines exhibit elevated lysosomal pH, causing T-DM1 to accumulate in the lysosome due to altered protein hydrolysis activity (27). Additionally, the deletion of lysosomal transporter protein expression, such as SLC46A3, can inhibit the release of the cytotoxic payload from the lysosome into the cytoplasm. Tomabechi et al. demonstrated that the deletion of SLC46A3 expression serves as a mechanism for innate and acquired resistance to ADCs carrying DM1 and PBD (81).

### 5.2 Drug delivery and other factors

In addition to the factors mentioned above, drug resistance mechanisms are partly attributed to issues with drug delivery. Hindrances may arise from antigen downregulation, loss of antigen expression, or antigenic mutations that affect their recognition by monoclonal antibodies. Reduced ErbB2 expression has been identified as an acquired resistance mechanism to T-DM1 in T-DM1-resistant cell lines (25). Moreover, tumors with higher heterogeneity and lower HER2 expression exhibit poorer drug efficacy due to limited permeability of the cytotoxic complex membrane (82). In addition, ADCs containing non-cleavable junctions can impact their efficacy. For instance, ARX788 with a maleimide junction and T-DM1 with a thioether junction may result in lower DAR, reduced payload numbers, and inferior anti-tumor effects (83). Notably, effective drug delivery can compensate for lower levels of antigen expression. Unlike conventional coupling approaches, ARX788 demonstrates advantages in PDX models with low HER2 expression levels by employing a site-specific engineered cysteine-based coupling approach (52). In future antibody design, the development of specific ADCs with high DAR using branched chain junctions or unpaired cysteines is anticipated. Additionally, optimization of antibody engineering and junctions is necessary. Promising progress has been made with dual paratope ADCs in advanced gastric cancer (NCT03821233), and ZW49, capable of targeting both HER2 and ZW25, has shown efficacy and low resistance in patients who have not responded to standard treatment with T-DM1.

In addition, the tumor microenvironment, recirculation of antigen-antibody complexes to the cell surface, and impaired drug release have been identified as factors influencing the efficacy of ADCs (84, 85).

## 6 Conclusion

Despite significant advancements in cytotoxic chemotherapy, immunotherapy, and targeted therapies in recent years, the long-term prognosis for patients with inoperable, recurrent, or metastatic gastric cancer remains unfavorable. The introduction of new drugs and the utilization of ADCs have brought about a transformative change in the treatment and prognosis of gastric cancer patients. However, there are certain limitations associated with their application. The impact of factors influencing antigen internalization and the heterogeneity of antigen expression on the effectiveness of ADCs remains unclear. Additionally, all existing cytotoxic drugs primarily target microtubule proteins or DNA/RNA, necessitating the re-optimization of chain strategies to enhance drug stability and site-specific coupling, thereby improving efficacy and safety. Furthermore, the development of drugs with diverse mechanisms of action, such as novel cytotoxic payloads, holds promise. Lastly, the future holds the potential for the discovery of novel predictive biomarkers that can aid in better defining patient subgroups with specific cancer characteristics.

## Author contributions

YZ, MZ and WK wrote the first draft of the manuscript. MZ and CL contributed to conception, review and editing. All authors contributed to the article and approved the submitted version.
